# Development of a Prototype Overground Pelvic Obliquity Support Robot for Rehabilitation of Hemiplegia Gait

**DOI:** 10.3390/s22072462

**Published:** 2022-03-23

**Authors:** Seunghoon Hwang, Seungchan Lee, Dongbin Shin, Inhyuk Baek, Seoyeon Ham, Wansoo Kim

**Affiliations:** 1 Department of Mechatronics Engineering, Hanyang University, 55, Hanyangdaehak-ro, Sangnok-gu, Ansan-si 15588, Gyeonggi-do, Korea; shwang45@asu.edu (S.H.); thetiru@hanyang.ac.kr (S.L.); sdbin225@hanyang.ac.kr (D.S.); bih0702@hanyang.ac.kr (I.B.); 2 Neuromuscular Control and Human Robotics Laboratory, Arizona States University, Tempe, AZ 85281, USA; 3 Department of Robotics Engineering, Hanyang University, 55, Hanyangdaehak-ro, Sangnok-gu, Ansan-si 15588, Gyeonggi-do, Korea; hcpretty99@hanyang.ac.kr

**Keywords:** rehabilitation robot, gait rehabilitation, pelvic motion support

## Abstract

In this work, we present the overground prototype gait-rehabilitation robot for using motion assistance and training for paralyzed patients. In contrast to the existing gait-rehabilitation robots, which focus on the sagittal plane motion of the hip and knee, we aim to develop a mobile-based pelvic support gait-rehabilitation system that includes a pelvic obliquity support mechanism and a lower-limb exoskeleton. To achieve this, a scissor mechanism is proposed to generate the paralyzed patient’s pelvic obliquity motion and weight support. Moreover, the lower limb exoskeleton robot is integrated with the developed system to provide the patient’s gait by correcting mechanical aids. We used computer-aided analysis to verify the performance of the prototype hardware itself. Through these methods, it was shown that our motor can sufficiently lift 100 kg of user weight through the scissor mechanism, and that the mobile driving wheel motor can operate at a speed of 1.6 m/s of human walking, showing that it can be used for gait rehabilitation of patients in need of a lower speed. In addition, we verified that the system drives the model by generating pelvic motion, and we verified the position controller of the integrated system, which supports the multi-degree motion by creating hip/knee/pelvic motion with a human dummy mannequin and systems. We believe that the proposed system can help address the complex rehabilitation motion assistance and training of paralyzed patients.

## 1. Introduction

According to the Centers for Disease Control and Prevention (CDC), in the United States, more than 79 million stroke cases occur each year [1], making strokes the leading cause of serious long-term disability. This negatively affects survivors’ natural gait. Due to lower ankle flexor weakness caused by strokes, stroke survivors cannot provide adequate push-off force during gait activities, and when weakness occurs in the hip abductor muscle, they have abnormal gait synptoms, such as circumduction gait and Trendelenburg gait [2,3]. Therefore research on gait rehabilitation for post-stroke patients is progressing rapidly [4].

Through these studies which assist correct rehabilitation and give walking assistance, paralyzed patients can become motivated to live and reduce the burden on national medical aid. A common method of rehabilitation for paralyzed patients is for them to experience normal walking [5]. Among the many medical devices developed for the rehabilitation of paralyzed patients, rehabilitation robots are being studied globally, and various types of rehabilitation robots have been developed.

Exoskeleton-type rehabilitation robots, such as Rewalk, provide good rehabilitation training [6]. However, the issues regarding the weight of these exoskeleton-type robots are important because they are worn by patients. The additional weight of the robot can lead to discomfort for the robot’s wearer. For reducing the number of actuators causing weight, the exoskeleton-type robots normally use actuators on the hip/knee joints, resulting in flexion/extension in the sagittal plane. These sagittal plane motions are dominant parameters of gait-rehabilitation robot design and control the assist force to patients. However, during gait-related activities, human joints have more than just one degree of freedom. To support other joint movements, additional actuators must be added to the robot’s system, which can result in problems of inertia and inconvenience for the human wearing it. Various methods have been proposed for solving these problems. A soft exosuit [7], which uses soft materials to reduce the weight of the exoskeleton, has become a recent trend. There are systems that use Bowden cables to transmit forces to the wearer’s hip and knee to provide assistance. A pneumatic system made by soft materials is also a proposed solution [8]. However, pneumatic actuators require an air tank to provide pneumatic pressure, which in turn results in additional weight for patients and restrictions on their mobility. These soft exosystems address the issue of wearability and weight to some extent, but they do not support the patient’s body weight, and additional external safety systems are thus required for ensuring safety during gait rehabilitation. Furthermore, these systems do not support all gait degrees of freedom.

However, the motions in other planes (e.g., pelvic tilt and obliquity motion) also play an important role in gait rehabilitation [9]. In particular, the pelvis is an important part of gait rehabilitation [3,10]. The movement of the pelvis plays an important role in moving a person’s center of mass (CoM) during walking and in maintaining the body’s balance [11]. Because of this importance, some pelvic-assistant robots support pelvic movements [12]. The PAM [13] and WalkTrainer [14] hold the patient’s pelvis for realizing body weight and motion support. The TPAD uses a different method from those used in other systems [15]. These systems are suitable for improving muscle strength by providing patients with a resistance force and support force for maintaining their balance, but they don’t facilitate their pelvic obliquity motion on the frontal plane during gait-related activities. In order to rehabilitate the patient’s motion accurately, the patient’s pelvic region must be rigidly restrained and attached directly to the patient’s pelvic region to assist the force and give correct pelvic support motion.

The rehabilitation space is also important to patients. Treadmill and overground gait rehabilitation both improve the patient’s gait [16]. However, some rehabilitation research results have shown that overground training improved gait ability to a greater extent in terms of parameters such as step length and gait-symmetric ratio [17]. However, not all patients can take overground gait training. The paralysis levels of patients are various; hence, the gait-rehabilitation approach is divided into two steps. First, the patient starts the training on the treadmill as rehabilitation. When the gait is improved, he/she moves to the next step of the overground gait training. This shows that the rehabilitation system should include both functionalities as the treadmill stationary system and an overground-type system to address the all-around gait rehabilitation. Therefore, some researchers have explored this issue by using a mobile-type system [18,19,20]. Seo [18] and Zou [19] have developed the drive wheel for autonomous movements. MTPAD [20] does not use the drive wheel, and this system thus needs the patients to apply force to move.

**Figure 1:**
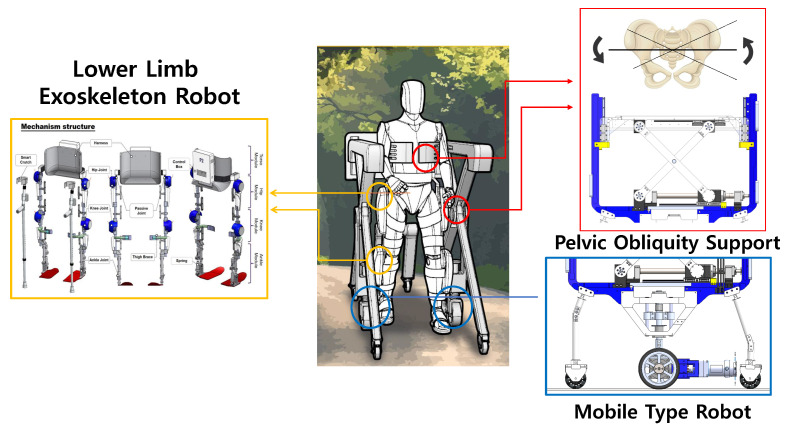
Concept of pelvic obliquity support robot. The red square represents the two scissor mechanisms located on each side of the patient. The scissor mechanisms support the pelvic frontal plane motion of the patient. The blue square represents the mobile-type robot. The drive wheel provides the system with mobility, thus allowing patients to undergo overground gait rehabilitation. The yellow square represents the lower-limb exoskeleton robot. The exoskeleton robot assists the hip and knee flexion/extension motion on the sagittal plane. The pelvic support robot and exoskeleton robot are integrated, and this integration facilitates patients with multiple degrees-of-freedom movement during gait rehabilitation.

In this study, we develop a mobile-type pelvic support robot for all-around gait rehabilitation, as shown in Figure 1. The developed system is composed of three modules: a mobile-type robot, a lower-limb exoskeleton robot, and a pelvic obliquity support mechanism. The mobile-type robot includes a driving wheel, which is actuated by a motor, and a shock-absorber mechanism to obtain the stability during overground rehabilitation. To provide the dominant motion in the sagittal plane (e.g., flexion/extension), the exoskeleton robot Walking Assist for Hemiplegia patients (WA-H) was integrated. It was developed in our previous work and evaluated for assistive motion in hip/knee flexion/ extension [21,22]. The pelvic obliquity support mechanism is developed to generate the pelvic obliquity motion in the patient’s frontal plane with two independently operated scissor mechanisms on each side of the patient. Moreover, the scissor mechanism is designed to provide body-weight support (BWS) including in the lower-limb exoskeleton robot. Consequently, the proposed mobile-type pelvic support robot can assist in multiple degrees-of-freedom motion for stationary rehabilitation as well as in overground rehabilitation for patients with various levels of paralysis.

The remainder of this paper is organized as follows. Section 2 presents the research background and aim of the design in terms of the parameters required to design a mobile-type pelvic support robot. Section 3 presents the detailed design, and Section 4 presents the evaluation of the scissor mechanism system, mobile wheel parts, and the position controller by CAE analysis and human mannequin dummy experiments. In the final section, the conclusions of this study and potential future work are discussed.

## 2. Design Objective and Requirements

### 2.1. Research Background: Hemiplegia Patients Gait

The majority of the paralysis caused by brain disease is hemiplegia, wherein the majority of the nerves are paralyzed, thus resulting in loss of motor ability in the sagittal plane. A hemiplegia patient who has lost the ability to walk with each leg will develop an abnormal gait to prevent the affected leg from being dragged along the ground while walking. One such abnormal gait is the hip-hike gait. It involves lifting the entire affected side of the body in a rotational motion toward the unaffected side based on the sagittal plane while using the spine force on the unaffected side and without using ankle force, such as by pushing off. The next most common gait of hemiplegia patients is the circumduction gait. Circumduction gait is a method of walking by rotating the affected leg toward the outside of the center of the body based on the transverse plane. Patients also exhibit Trendelenburg gait symptoms. The Trendelenburg gait is a phenomenon wherein the gluteus medius supporting the pelvis and femur is weakened, and the pelvis supporting the weight falls out to the side during walking. The hemiplegia patient is in a weak state on the affected side of the gluteus medius during walking. Therefore, if the patient continues to support the weight on the unaffected side, this phenomenon occurs and gradually worsens and has a negative effect on the skeleton of the lower limb [23,24,25].

### 2.2. Design Objective for Body Weight Support, Pelvic Obliquity Motion Support, and Multiple Degrees-of-Freedom Movements

While walking, a repulsive force greater than the body weight of the person is exerted from the ground. These repulsive forces differ from one person to another, and the gait characteristics of paralyzed patients also differ. At the same time, as an application of the ground reaction force, the movement of the pelvis also differs in each paralysis patient. The movement of the CoM, which comprises the human weight, moves up to 6 cm during walking, and at the same time, the pelvic movement in the frontal plane also exhibits a movement of approximately 5${}^{\circ }$. To take into consideration these pelvic movements, in this study, a scissor mechanism was proposed to assist BWS and pelvic support.

The scissor mechanism can amplify forces by using the principle of levers and is used in many mechanical devices and tools. By using the scissor mechanism, heavy objects can be lifted with a small force acting on the motor. The human body weight varies from approximately 50 kg or less to 100 kg or more, so the robot system is required for up to 100 kg, and more force is required to assist and support a heavier body. Support for pelvic movement also requires strength. Therefore, we can expect good results from the application of scissor mechanisms to new types of rehabilitation robots. We placed two scissor mechanisms on the left and right sides of the patients to simultaneously satisfy the concept of BWS and pelvic obliquity assistance.

**Figure 2:**
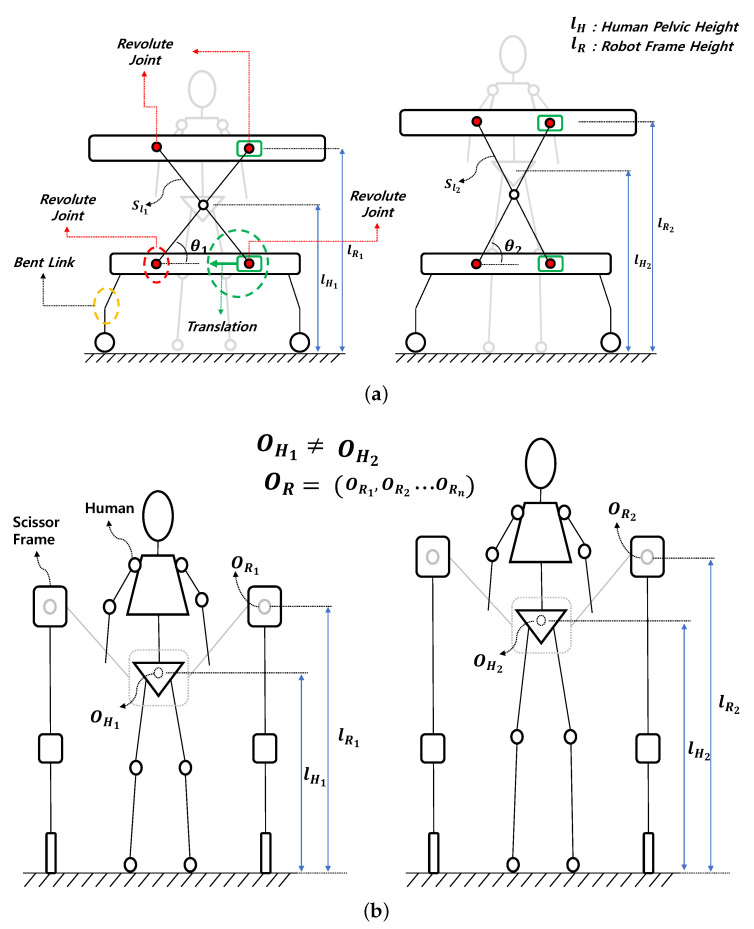
Conceptual design of the scissor mechanism. (**a**) The scissor mechanism’s conceptual design viewed from the side. Scissor mechanism connected with revolute joints (red circles) and two wheels with links supporting each systems’ weight (orange dottted circle). (**b**) Scissor mechanism’s conceptual design viewed from the front, which shows how our system can adjust to various human pelvis positions.

In Figure 2a, the scissor mechanism is illustrated. The wheel is located at the bottom of the robot and makes contact with the ground, and the bent link (orange circle in Figure 2a) supports the lower frame of the scissor mechanism. All red circles indicate the revolute joints that are connected to links of the scissor mechanism. The green circle and square indicate the translation joint and the link connected to the revolute joint. The two links of the scissor mechanism are fixed with a revolute joint and support the upper frame.

Figure 2b presents the scissor mechanism viewed from the front, and it shows that the scissor mechanism is placed on both sides of the person. A person is positioned in the middle of the scissor mechanism arranged on both sides, and the scissor mechanism is fixed to the harness that the person wears around their pelvis.

The height of a person cannot be standardized, because it is affected by so many different variables such as gender, age, etc. Therefore, the location of a person’s pelvis has different values for each person, as shown in Figure 2b ($O_{H_{1}}\neq O_{H_{2}}$). $l_{H}$ in Figure 2 represents the distance from the ground to the center of the human pelvis, and $l_{R}$ is the position of the upper frame of the scissor mechanism from the ground. An adjustable pelvic position that everyone can use is an important factor in the development of exoskeleton robots or rehabilitation robots. Our system has the advantage of the mechanism being able to efficiently respond to the different heights of different people because the scissor mechanism can move vertically, as shown in Figure 2a,b.

**Figure 3:**
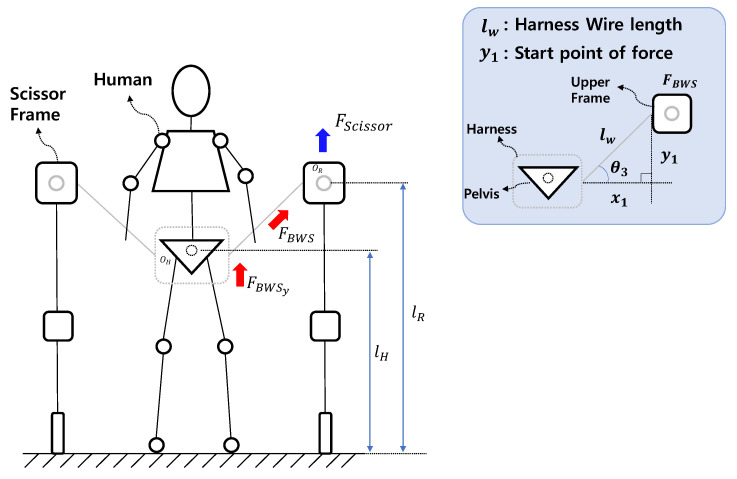
The scissor mechanism applies the BWS function for paralyzed patients. The direction of force action is shown.

To generate BWS force on the patients, the upper frame of the scissor mechanism needs to move upward when the harness is pulled. Figure 3 illustrates the state of $F_{BWS}$. $l_{w}$ represents the length of the wire connected to the harness worn by a person, and the upper frame of the scissor mechanism, and the wire length is constant. $x_{1}$ is the distance between the harness, and the upper frame of the scissor mechanism. $y_{1}$ is the minimum height required for generating $F_{BWS}$. The height of the upper frame of the scissor mechanism can be expressed by using the following equations:

(1) $$\cos\theta_{3}=\frac{x_{1}}{l_{w}},$$

(2) $$\sin\theta_{3}=\frac{y_{1}}{l_{w}},$$

(3) $$y_{1}=l_{w}\sin\theta_{3},$$

(4) $$\mathrm{and}\;l_{R}=y_{1}+l_{H}.$$

The relationship between $x_{1}$ and $l_{w}$ can be represented by the cosine relation, and the relationship between $y_{1}$ and $l_{w}$ can be represented by the sine relation. Finally, $y_{1}$ can be expressed via Equation (3). The height from the ground of the upper frame of the scissor mechanism must be the sum of the pelvic positions of the person $l_{H}$ and $y_{1}$, as shown in Equation (4).

$F_{BWS}$ is the BWS force and $F_{Scissor}$ is a force generated by driving the scissor mechanism, which acts in a direction perpendicular to the ground. The full amount of $F_{Scissor}$ caused by the scissor mechanism is not fully delivered to the person because the wire connected to the human pelvis and the upper frame is not parallel to the driving direction of the scissor mechanism. Thus, the $F_{Scissor}$ is transmitted to a person in a state converted by $\theta_{3}$, which can be represented by $F_{BWS}$. The $F_{BWS}$ generated by $\theta_{3}$ results in a force in two directions for the patient. At this time, the pure force that compensates for the human weight, i.e., BWS, can be represented by $F_{BWS_{y}}$, which corresponds to the y direction of $F_{BWS}$.

If $F_{BWS_{y}}$ is a pure force that lifts the patient’s body, $F_{BWS_{x}}$ is the force that pulls the patient toward the robot frame. The greater $F_{BWS_{x}}$ is, the more the body moves from side to side along the frontal plane when the patient is walking, and thus, appropriate walking cannot be realized. To reduce the negative force $F_{BWS_{x}}$, the distance between the robot and the x-axis, $x_{1}$, must be reduced. The force of $F_{BWS_{y}}$ is amplified as the length of $x_{1}$ is reduced.

**Figure 4:**
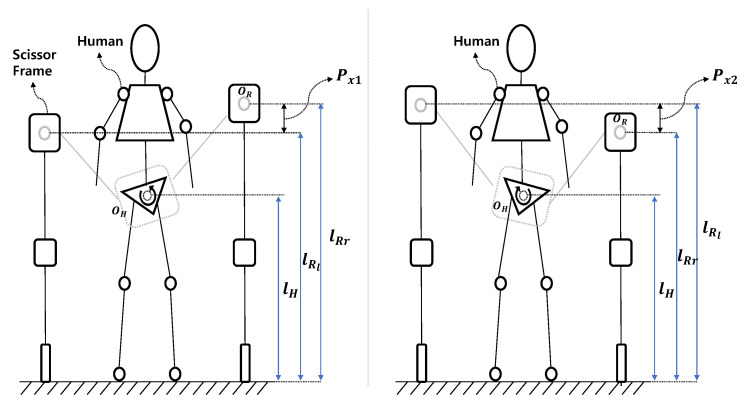
The appearance of using the scissor mechanism to generate the pelvic motion of paralytic patients. (**Left**) If right mechanism move upward and left mechanism move downward, system make counter clockwise pelvic obliquity motion. (**Right**) If left mechanism move upward and right mechanism move downward, system make clockwise pelvic obliquity motion.

The motion support for the pelvis can be satisfied by using the scissor mechanism. Figure 4 shows that the upper frames of the scissor mechanism on the left and right sides are different. The human pelvic movement in the frontal plane comprises the obliquity motion. Paralyzed patients are unable to perform pelvic obliquity motions by themselves. Therefore, they have difficulty in muscular exercise or rehabilitation exercise. In order to support pelvic motion during rehabilitation exercise in paralyzed patients, the scissor mechanism drives their motor and make the right/left mechanism height difference for generating a rotating motion.

In Figure 4, $l_{R_{l}}$ is the height from the ground of the upper frame of the scissor mechanism located on the left side of a person and $l_{R_{r}}$ is that of the upper frame located on the right side of a person. If $l_{R_{l}}$ and $l_{R_{r}}$ have different values, this make their difference length $P_{x_{1}}$. Through this length difference between right and left, if patients need to support rotational motion in the counterclockwise direction of the human pelvis, the scissor mechanism is activated such that the upper frame on the right side moves upward and the left frame moves downward. Conversely, if a patient needs to rotate it clockwise, the scissor mechanism is activated such that the upper frame on the left side moves upward and the right side moves downward. The range of motion of the scissor mechanism for supporting the pelvic movement is based on a human range of motion of approximately −5${}^{\circ }$ to 5${}^{\circ }$. Moreover, by using the scissor mechanism, the balance of the pelvis can be adjusted when patients exhibit Trendelenburg symptoms. In this paper, we develop a new rehabilitation robot to lift the weight of a person instead and to satisfy the efficient function of supporting the pelvic motion at the same time by this scissor mechanism.

Our system is focused on pelvic movements, and we also take care to account for the dominant movement, which may be hip/knee flexion/extension by using our exoskeleton-type robot. We integrate the exoskeleton robot with a pelvic support robot. The robot can support more than patients’ gait motion in the 2D plane, because the pelvic robot supports frontal plane motion and the exoskeleton robot supports sagittal plane motion simultaneously.

### 2.3. Design Objective for Overground Gait Rehabilitation

Rehabilitation robots, such as Rewalk [26] and Lokomat [27] are different types of robots. Lokomat is capable of rehabilitation treatment in a stable condition, but it has limitations. Patients take rehabilitation only by facilitating training with a treadmill. Overground training provides the patient with a better therapeutic effect in their rehabilitation treatment than with a treadmill [17,28]. The Vector [29] can provide overground training, but it requires a large space with a guidance system on the ceiling. In the case of Rewalk, this exoskeleton-type robot can also provide overground rehabilitation; however, it cannot provide BWS to patients. Therefore, we developed a mobile-type rehabilitation robot for overground gait rehabilitation with no space limit in addition to the faciliation of BWS.

## 3. Pelvic Obliquity Support Robot Design

### 3.1. Detailed Design of Scissor Mechanism for BWS and Pelvic Obliquity Support

**Figure 5:**
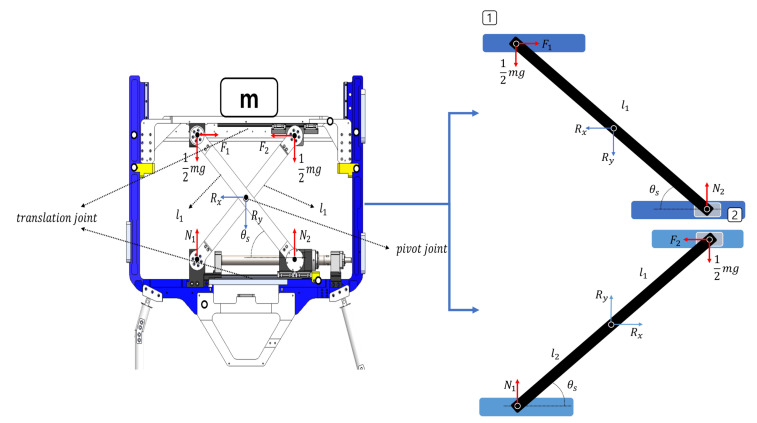
Relationship of forces generated by scissor mechanism when a certain mass is present. $l_{1}$ is the scissor mechanism link length and $\theta_{s}$ is the scissor mechanism angle. Number 1 box and 2 box show the scissor mechanism link separately and derive free body diagram for showing force relationship.

The scissor mechanism has been applied in various studies in various forms and is one of the mechanisms that has been studied [30]. The structure of the scissor mechanism to be applied in this study is shown in Figure 5. The two scissor links are bounded by a pivot joint. The upper and lower frames are fixed via a hinge joint, and one side was bound with a translation joint. If the system doesn’t have a translation joint, the scissor mechanism can’t move upward or downward and remains static.

As shown in Figure 5, when any object exists in the upper frame, the mass of the object is transferred to each scissor link through the upper frame. The new type of rehabilitation robot developed in this study comprises a form that supports the body weight of the patient, and the object can be assumed to be the weight of the patient. When such masses exist, forces are transmitted to the hinge joint of each scissor link through the upper frame, and the force corresponds to half the mass. The value of the force is half the mass when it is assumed that two hinge joints have the same distance based on the pivot joint. If the distance between two hinge joints is different, the ratio of the mass added to each hinge joint also changes.

The force generated in each upper hinge joint by any mass *m* generates a reaction force in the lower hinge joint bound to the lower frame through the scissor link. The force generated in each hinge joint of the lower frame is changed by the force generated in the hinge joint of the upper frame and by the state angle of the scissor $\theta_{s}$ which is the angle between one scissor link and one frame. The four values of $\theta_{s}$ obtained are based on the conditions that the lengths of the two scissor links are the same and that there is a pivot joint in the middle of the link, and the value of the angle is the same. The value of $\theta_{s}$ changes with the translation joint, which affects forces $F_{1}$ and $F_{2}$.

If there is no bound to the translation joint when there is any mass *m* in the scissor mechanism, as shown in Figure 5, the upper frame falls freely and comes into contact with the lower frame. Forces $F_{1}$ and $F_{2}$ are necessary to maintain the position of the scissor mechanism in any $\theta_{s}$ state, as shown in Figure 5. Therefore, $F_{1}$ and $F_{2}$ are the forces that maintain the scissor mechanism in the $\theta_{s}$ state when the object *m* is present. The forces of $F_{1}$ and $F_{2}$ are the same under the condition that each scissor link has the same length and there is a pivot joint in the middle of the link, as in the condition of $\theta_{s}$.

$R_{x}$ and $R_{y}$, which are the reaction forces generated in the pivot joint, are presented. $R_{x}$ and $R_{y}$ generated in the pivot joint are evidence that scissor mechanisms are bound to each other for any mass *m* and distribute uniform forces. If there is no pivot joint, there will be no reaction force $R_{x}$ and $R_{y}$, and each scissor link will distribute different forces for mass *m*. The force equilibrium equation of Figure 5 (right upward) is as follows:

(5) $$\sum F_{x}=-R_{x}+F_{1}=0,$$

(6) $$\sum F_{y}=-R_{y}+N_{2}-\frac{mg}{2}=0,$$

(7) $$\mathrm{and}\;\sum \mu =\frac{mg\cos\theta_{s}l_{1}}{4}-\frac{F_{1}\sin\theta_{s}l_{1}}{2}+\frac{N_{2}\cos\theta_{s}l_{1}}{2}.$$

We can then define the force equilibrium equation in Figure 5 (right downward), as

(8) $$\sum F_{x}=R_{x}-F_{2}=0,$$

(9) $$\sum F_{y}=R_{y}+N_{1}-\frac{mg}{2}=0,$$

(10) $$\mathrm{and}\;\sum \mu =-\frac{mg\cos\theta_{s}l_{1}}{4}+\frac{F_{2}\sin\theta_{s}l_{1}}{2}-\frac{N_{1}\cos\theta_{s}l_{1}}{2}.$$

The relationship between the forces generated in each scissor link can be defined through the Equations (5)–(10). The forces generated by the two scissor links are related and similar. The following relational expression can be additionally made through the defined Equations (5)–(10).

(11) $$-R_{x}+F_{1}=-R_{x}+F_{2},$$

(12) $$F_{1}=F_{2}.$$

In the case of the force along the x-axis direction of each scissor link, the magnitude of forces generated in the pivot joint forces $F_{1}$ and $F_{2}$ are the same. The following relational expression can be obtained by using Equations (6) and (9), as shown in Equation (11):

(13) $$-R_{y}+N_{2}-\frac{mg}{2}=-R_{y}-N_{1}+\frac{mg}{2},$$

(14) $$N_{1}+N_{2}=mg,$$

(15) $$\mathrm{and}\;N_{1}=N_{2}=\frac{mg}{2}.$$

The following relational expression can be obtained by using the concepts of Equations (7) and (15).

(16) $$0=\frac{mg\cos\theta_{s}l_{1}}{4}-\frac{F_{1}\sin\theta_{s}l_{1}}{2}+\frac{mg\cos\theta_{s}l_{1}}{4},$$

(17) $$-\frac{F_{1}\sin\theta_{s}l_{1}}{2}=-\frac{mg\cos\theta_{s}l_{1}}{4}-\frac{mg\cos\theta_{s}l_{1}}{4},$$

(18) $$-\frac{F_{1}\sin\theta_{s}l_{1}}{2}=-\frac{l_{1}mg\cos\theta_{s}}{2},$$

(19) $$\mathrm{and}\;F_{1}=\frac{l_{1}mg}{l_{1}tan\theta_{s}}.$$

To obtain the value of $F_{1}$, the term containing $F_{1}$ was moved to the left side to obtain Equation (17). In addition, the term on the right side was integrated into the length of the scissor link to obtain Equation (18), and finally, the force $F_{1}$ for maintaining the position of the scissor mechanism was defined.

We simulated the scissor mechanism by using MATLAB 2020 under the following conditions and by using the previous relational expressions in Table 1.

**Figure 6:**
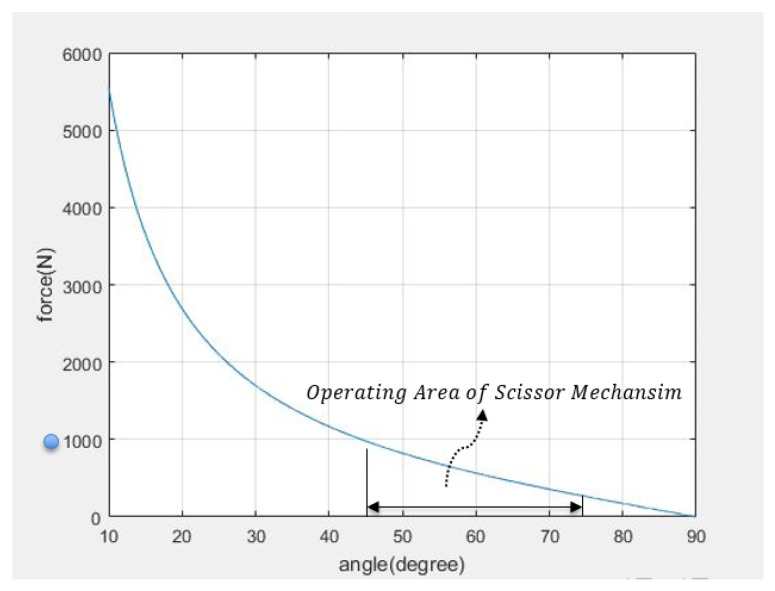
The required $F_{1}$ value for scissor static state by $\theta_{s}$. If scissor mechanism drive on operation area, scissor mechanism use the force lower than 1000 N (blue point) for supporting 100 kg person.

$\theta_{s}$ cannot reach $0^{\circ }$ because the structure of the scissor mechanism and also the maximum angle cannot exceed $90^{\circ }$. The weight applied in the direction of gravity in the upper frame of the scissor mechanism is set as 100 kg owing to the safety rate of the mechanism itself. One of the goals of the new type of rehabilitation robot is BWS, which is a concept according to which assistance can be provided to help paralyzed patients support their body weight. The weight of paralyzed patients varies, and the range also differs accordingly. In consideration of this, we set the performance of the scissor mechanism to support the weight of paralyzed patients weighing more than 100 kg. The simulation results are presented in Figure 6 where $\theta_{s}$ = $0^{\circ }$, and a force of 5000 N or greater is required. Therefore, it is not good in terms of the efficiency of a vertical lifting mechanism. This phenomenon is observed from $\theta_{s}$ = $10^{\circ }$ and continues to $\theta_{s}$ = $45^{\circ }$. The reason for this phenomenon is the essential single problem of the scissor mechanism, and the link angle of $10^{\circ }$∼$45^{\circ }$ of the scissor mechanism can be considered as a single driving area.

When the link angle of the scissor mechanism exceeds $45^{\circ }$ through the singular driving region, it is possible to maintain the posture for $\theta_{s}$ by using a force of less than 1000 N. Therefore, if the link angle of the scissor mechanism is $45^{\circ }$ or greater, the efficient side of the force is good because the arbitrary mechanism can lift the load with a force less than 1000 N, which is the force required to lift the load vertically. As the value of $\theta_{s}$ gradually increases, the force $F_{1}$ required to lift the weight is reduced. From the state of $\theta_{s}$ = $60^{\circ }$ or greater, our objective can be realized by using a force of approximately half of 1000 N. The condition should be that less than a force of 1000 N is required to lift a 100 kg weight vertically, and it must be in an area where the scissor mechanism can be driven structurally,

(20) $$\theta_{s}=45^{\circ }∼65^{\circ }.$$

The system is configured so that the ball screw included in the scissor mechanism can have good efficiency by amplifying the power of the motor. The normal and stall torque of the motor used in the scissor mechanism is 316 mNm, 6110 mNm, respectively. The maximum speed of the motor is 10,000 rpm, and as the load increases, the usable RPM decreases. The torque constant of the motor is 42.7 mNm/A, the required torque of the scissor mechanism to maintain the 100 kg is 124.65 mNm, and an amount of the current of about 3 A is required. The voltage applied to the motor is 48 V. Our system total weight about 120 kg. Most of the mechanical materials for constituting the robot and scissor mechanism used the AL60 series for weight efficiency, and this part needs to be strong, preventing fatigue failure. It was manufactured by using SM45C series material.

**Figure 7:**
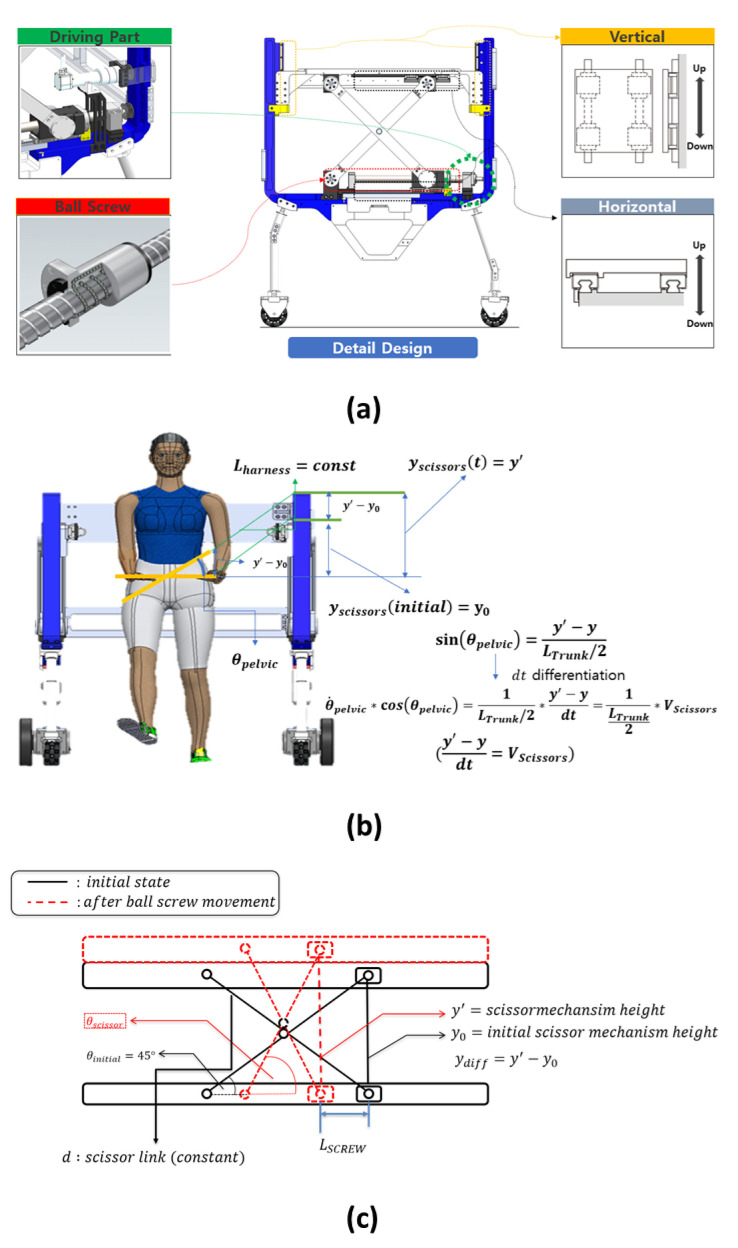
(**a**) The detailed design of the scissor mechanism. The driving part (green and red parts in (**a**) consists of the BLDC motor and ball screw. If the BLDC motor rotates, the ball screw changes the motor rotation to linear motion. Then, the bottom of the scissor mechanism moves in the horizontal direction through the LM guide (gray part in (**a**), and the top of the scissor mechanism moves in the vertical direction through the LM guide (yellow part in (**a**). As a result, this vertical movement facilitates the BWS of the patient and pelvic obliquity movement along the frontal plane. (**b**,**c**) The kinematic model. (**b**) The modeling of the ball screw to the scissor mechanism. (**c**) The scissor mechanism for the human pelvic obliquity movement.

As in Figure 7, pelvic-supporting ball screws of the B-system linearly move 36 mm when the motor rotates $360^{\circ }$. This relation can be derived as

(21) $$L_{SCREW}=36a\;\mathrm{mm}\left(a=\frac{\theta_{sys}}{360^{\circ }}\right),$$

where *a* means number of rotations, $L_{Screw}$ is the linear displacement, and $\theta_{sys}$ is the scissor-mechanism motor angle when the motor is being driven. To calculate the linear velocity of the ball screw $V_{SCREW}$ in the scissors mechanism, we differentiate Equation (21) by time. We can thus obtain Equation (22),

(22) $$V_{SCREW}=36a/dt=36\left(\frac{\dot{\theta_{sys}}}{360^{\circ }}\right).$$

Then, we can calculate that the up and down mechanism $\theta_{s}$ is 10${}^{\circ }$, and a force of 5000 N or greater must be generated at $F_{1}$ to lift 100 kg. However, to maintain the link angle of the scissor mechanism $\theta_{s}$ at 10${}^{\circ }$, a greater velocity of the scissor mechanism is required by using $V_{SCREW}$. Figure 7c presents a side view of the scissor mechanism. The black line represents the initial state of the scissor mechanism, and the red dotted line occurs after the motor driving state. The constant *d* is the scissor link, and we can express the Pythagorean theorem as Equation (23),

(23) $${\left(y_{diff}+\frac{d\sqrt{2}}{2}\right)}^{2}+{\left(\frac{d\sqrt{2}}{2}-L_{SCREW}\right)}^{2}=d^{2}.$$

To calculate the up and down velocity of the scissor mechanism, we differentiate Equation (23) by time,

(24) $$\begin{array}{c} V_{SCISSORS}\left(y_{diff}+\frac{d\sqrt{2}}{2}\right)+V_{SCREW}\left(\frac{d\sqrt{2}}{2}-L_{SCREW}\right). \end{array}$$

After representing $V_{SCISSORS}$ in Equation (24), this term can be expressed as follows,

(25) $$V_{SCISSORS}=V_{SCREW}\frac{\left(L_{SCREW}-\frac{d\sqrt{2}}{2}\right)}{\left(y_{diff}+\frac{d\sqrt{2}}{2}\right)}.$$

The following is a relational expression between the pelvic angle and the scissor mechanism. A few assumptions are defined before expressing the expression. It is assumed that the length of the harness connecting the human pelvis and the scissor mechanisms is constant. As shown in Figure 7b, we can express the relationship between the human pelvic obliquity angle and the scissor mechanism differentiation as

(26) $$sin\left(\theta_{pelvic}\right)=\frac{2y_{diff}}{L_{trunk}}.$$

The differentiation of Equation (26) is expressed as follows:

(27) $$\begin{array}{c} \dot{\theta_{pelvic}}\cos\left(\theta_{pelvic}\right)\frac{2V_{SCISSORS}}{L_{trunk}}\left(\frac{y_{diff}}{dt}=V_{SCISSORS}\right). \end{array}$$

On combining Equations (25) and (27), the relationship between the linear velocity of the ball screw of the scissor mechanism and the human pelvic angular velocity can be obtained, and the relationship between the linear displacement of the ball screw and the pelvic angle can be expressed as

(28) $$\begin{array}{c} \dot{\theta_{pelvic}}\cos\left(\theta_{pelvic}\right)=\frac{2V_{SCREW}\left(L_{SCREW}-\frac{d\sqrt{2}}{2}\right)}{L_{trunk}\left(y_{diff}+\frac{d\sqrt{2}}{2}\right)}. \end{array}$$

By using Equation (28), we can calculate the desired scissor mechanism motor position and the velocity for the desired human pelvic obliquity angle and pelvic obliquity angular velocity. As previously mentioned, Equation (28) expresses the connection between the scissors mechanism and human pelvic obliquity movements. In order to use Equation (28) to calculate the desired scissor mechanism motor position and velocity, we should plan the desired human pelvic motion.

### 3.2. Detailed Design of Mobile Type Robot for Overground Gait Rehabilitation

**Figure 8:**
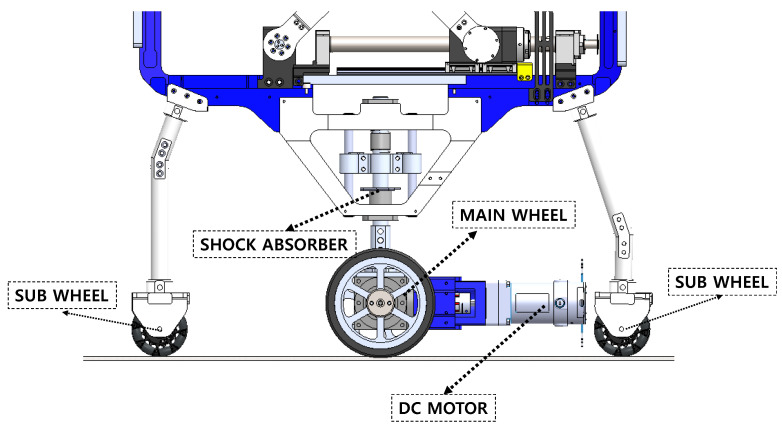
Detailed design of the mobility part. The front and back sides of the robot have sub-wheels. The sub-wheels were of the omni-wheel type. Omni-wheels can allow a system to move in all directions (e.g., rotation). Omni-wheels do not have an actuator. The objective of the sub-wheel is direction guidance and to avoid disturbing the main wheel drive. The main wheel is located at the middle of the robot. The main wheel comprises a DC motor that generates the driving force to realize the mobility of the robot and patients. The mobility part also comprises a shock absorber. This part allows the robot to travel over various terrains during overground gait rehabilitation.

We design the mobility of the rehabilitation robot such that it can follow patients. The mobility part consists of main wheels and sub-wheels. The sub-wheel is located on the back and front of the robot, as shown in Figure 8. The sub-wheel is of an omni-wheel type and is not driven by an actuator. The omni-wheel enables the robot to drive in rotation as well as in a straight line. The sub-wheel can carry a load of 123 kg, and it can thus hold the weight of the robot and patient. In Figure 8, we present the design of the main driving wheel. The main driving wheel is not simply driven, and the main wheel always makes contact with the ground for safety. Therefore, the shock absorber is combined with a linear bush and spring to perform vertical translation motion and for stiffness.

The mathematical relation formula for selecting a drive motor to move the entire frame of a newly developed rehabilitation robot in accordance with the walking speed of a paralyzed patient is as follows:

(29) $$\tau_{c}=\frac{\mu_{c}mD}{4}.$$

Equation (29) presents a constant torque that occurs when a certain object acts as a load to be driven by the wheel on which it is mounted. $\mu_{c}$ is a coefficient generated when the surface of the wheel and the ground of the floor are in contact, and in the calculation, the contact coefficient of rubber and concrete is set as 0.1. *D* is the diameter of the driving wheel. In the case of *m*, we assumed that 250 kg of weight on one side is transmitted through the human and robot frames.

As a result of the calculation of Equation (29), the torque through the driving wheel for the robot to move at a constant speed when a load of 250 kg is applied is calculated as 1.5 Nm.

(30) $$f_{a}=\frac{t_{h}}{\pi D},$$

(31) $$J_{a}=\frac{mD^{2}}{8},$$

(32) $$\tau_{a_{1}}=\frac{2\pi f_{a}J_{a}}{gt_{a}}.$$

The torque required for uniform motion and the torque for the acceleration motion of the robot were calculated as shown in Equation (32). Equation (30) is a calculation used for determining whether a rotation should be made within seconds of the driving wheel designed to match a person’s walking speed. The walking speed of paralyzed patients is slower than that of the general population, but in this formula, it is set as 1.6 m/s, which is the average walking speed of the general public. The denominator of Equation (30) is the circumference of a circle that is generated when the driving wheel rotates for 1 s. Thus, $f_{a}$ is a constant speed of the desired wheel, and the calculation result is determined as 2 rps.

Equation (31) is the load inertia moment for any load, and we set the load *m* as 250 kg, for a constant torque calculation. $J_{a}$ is calculated as 1.95 kgm${}^{2}$. Finally, we calculated Equation (32), which is the torque required for acceleration based on Equations (30) and (31) and set the target acceleration time $t_{a}$ as 0.1 s. The reason for setting the acceleration time ta as 0.1 s is to allow the robot to overcome the distance error with the robot that occurred when the human started walking as soon as possible. The calculated $\tau_{a}$ is calculated as 25.5 Nm:

(33) $$\tau_{a_{2}}=\tau_{c}+\tau_{a_{1}}.$$

The torque of the acceleration interval for driving the new type of mobile robot developed in this study is that presented in Equation (33), which is the sum of the constant torque and acceleration torque. We selected a motor for driving the driving wheel based on the calculated values. The selected motor generates at least 25 Nm of torque and performs at a speed of 2 rps or greater.

### 3.3. Detailed Design of the Lower-Limb Exoskeleton Robot for Multiple Degrees of Freedom

**Figure 9:**
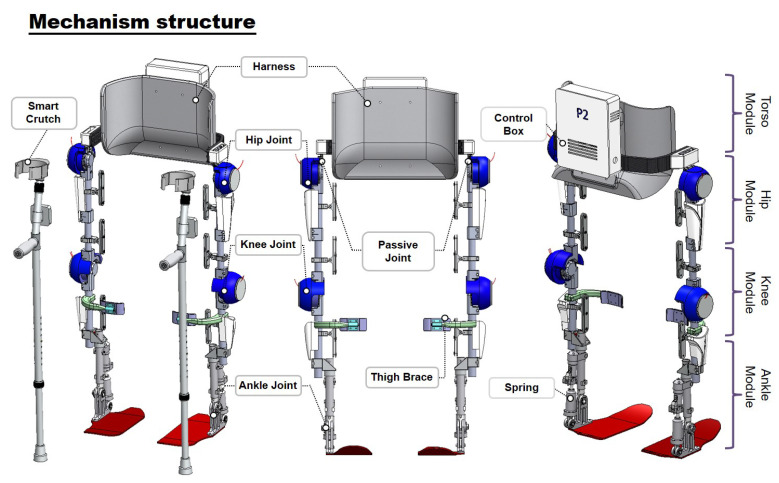
Our exoskeleton robot has four active and two passive joints in the sagittal plane. The four active joints assist the hip and knee flexion and extension motion. The two passive joints can realize free movements on ankle plantar flexion and dorsi flexion. In the frontal plane, for hip abduction and adduction movements, we design one passive joint for both the right and left hip.

For this study, we used the walking assist robot for hemiplegia patients (WA-H) that we developed in our previous research in Figure 9. The robot assists the sagittal plane motion by using four motors, two each on hip joints and knee joints. The ankle has a passive joint using a spring to prevent foot-drop and to assist dorsi flexion, and the robot has a passive joint for hip-joint abduction and adduction movement to trace the wearer’s movement without causing inconvenience. Therefore, the WA-H robot has a total of eight DOF joints consisting of four active joints and four passive joints for gait training. All joints are designed to prevent user injuries from operating the robot over the human range of motion by H/W stoppers and motors S/W limit. Each active joint has a passive mode in which the wearer can move freely by assisting only gravity compensation and harmonic drive friction compensation torque, and an active mode in which the robot assists force to the user by using the training gait pattern. In addition, the robot can possibly generate gait patterns by measuring the user pose and generating a walking pattern corresponding to the user pose [21]. We even verified the effect of the gait rehabilitation by using our robot with hemiplegia patients through previous studies [22].

## 4. System Verification and Result

### 4.1. CAE Verification of Scissor Mechanism and Mobile Driving

**Figure 10:**
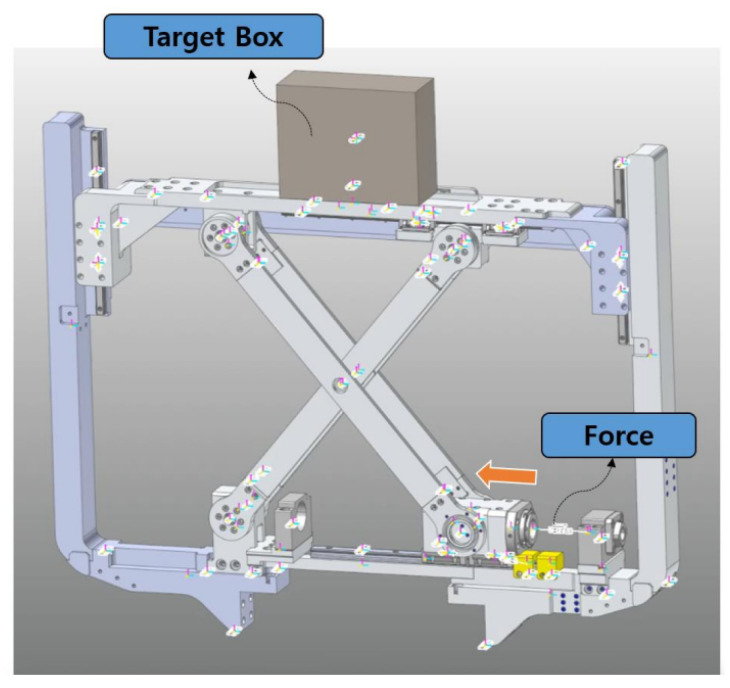
CAE analysis environment based on 3D design for scissor mechanism verification.

In Section 3.1, we derived the characteristic of the scissor mechanism. To verify the system, we performed computer-aided engineering (CAE) analysis. Because driving of the scissor mechanism does not affect the lower driving wheel part, we included the necessary parts, components, and constraints condition that affect the scissor mechanism in CAE models, as shown in Figure 10.

The CAE model includes frames that can fix the scissor mechanism and the upper frame that moves up and down through the scissor mechanism. In addition, a box-shaped object was added on the upper frame instead of the body-weight support force generated by the scissor mechanism. The weight of the box object is 50 kg.

In the lower part of Figure 10, the position of the force generated for CAE can be confirmed. The force value generated by the ball screw is 500 N and give to system during 1 s. The initial angle of the scissors mechanism is $45^{\circ }$. The total time for analysis is 3 s, and the analysis step size is 100.

**Figure 11:**
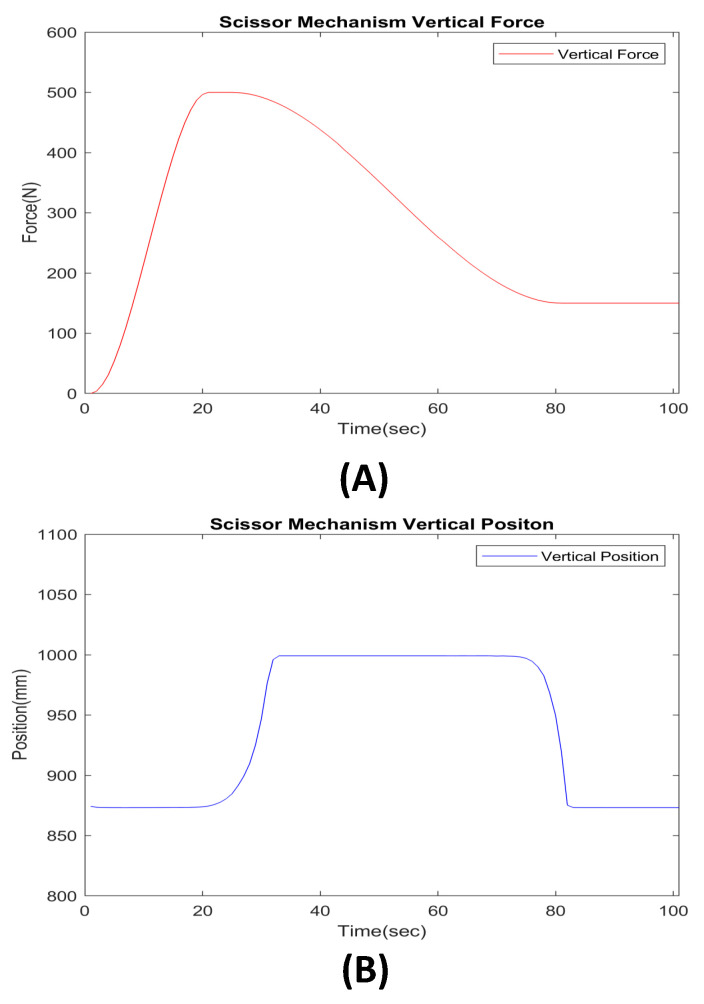
Scissor mechanism CAE analysis results data. (**A**) The vertical force was generated by the scissor mechanism for lifting a 50-kg object (0 to 500 N). (**B**) Scissor mechanism vertical position. The position of the object rises as the scissor mechanism generated force in the direction of gravity (initial position 850 mm to final position 1000 mm).

As shown in Figure 11 our mechanism generated a force up to 500 N in the vertical direction through the ball screw to lift a weight of 50 kg in the direction of gravity, and (B) shows that the object has risen in the direction of gravity. According to the configuration for multi-body dynamics (MBD) CAE, in addition to 50-kg objects, the weight of the scissor link, the weight of the ball screw, and the top plate are added as loads, and fraction elements of additional system elements are added. Therefore, the applied force of 500 N overcomes forces other than lifting a 50-kg object, and it can be proved to be efficient. In conclusion, our system uses two scissor mechanisms on both sides. Our system can perform BWS by lifting the weight of patients under 100 kg and can generate pelvic motion motion of patients through this vertical force.

**Figure 12:**
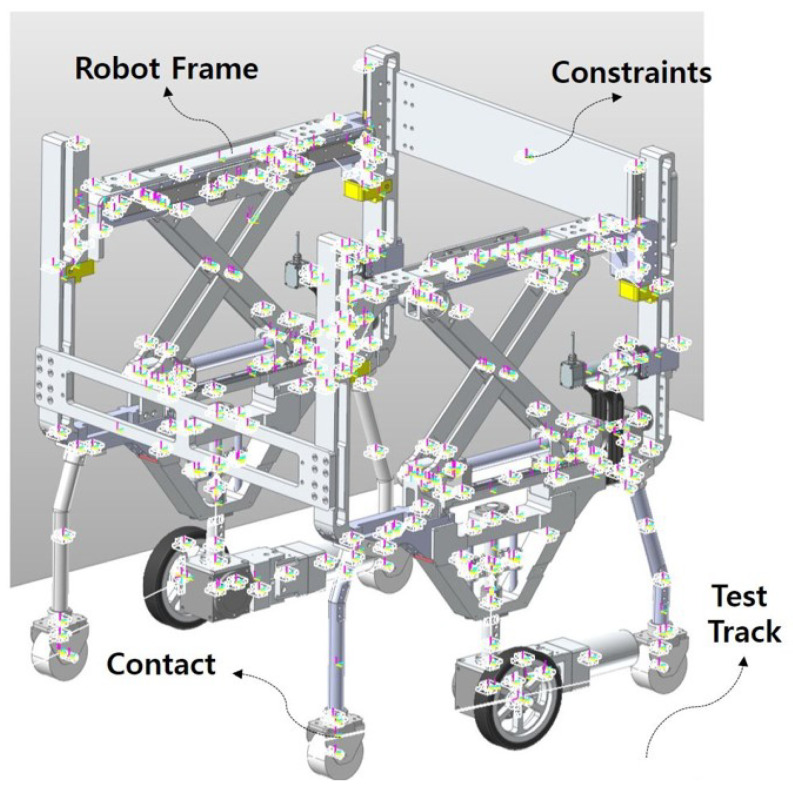
CAE analysis environment based on 3D design for the mobile part of the system. Contact environment friction coefficient = 1 and damping coefficient = 10. The system material set Al60 as the same as the real system.

We also verified the driving capacity of the entire system based on the torque value of the driving wheel. The CAE model environment is in Figure 12. We set constraints and contact conditions for all parts of the system. The dynamic friction coefficient value was set at 1 under the contact conditions for the wheel and ground and the damping coefficient value was 10. We set the material of all robot parts used in the analysis to Al60 and steel same as actual system.

We analyzed the operation of the mobile driving part too. The goal of the driving analysis is to evaluate whether the system can drive at the desired velocity in the simulation with the input wheel motor torque value. The step time for analysis is the same as the scissor mechanism analysis. We analyzed the forward directions of the system, and we set our system’s desired velocity value as 1.6 m/s.

**Figure 13:**
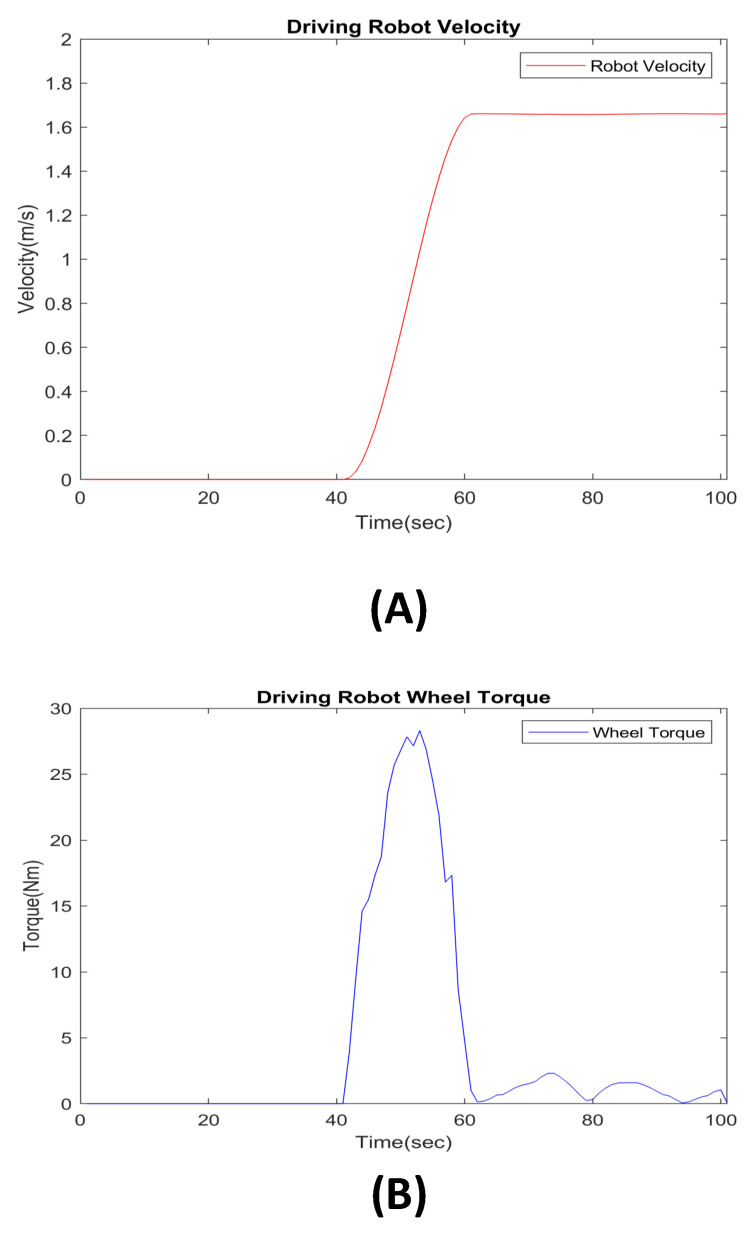
Driving CAE analysis result data. The CAE results show wheel torque and the angular velocity value for following user walking speed (1.6 m/s). (**A**) System velocity. (**B**) System wheel torque data.

In Figure 13B, we show the torque plot for the driving system with our desired system velocity. The torque value for 1.6 m/s velocity is lower than 30 Nm. Because the motor of our system can generate torque up to 70 Nm, it can follow a walking speed of 1.6 m/s, indicating that the system can move at greater than 1.6 m/s. Therefore, our system can cover the walking speeds of various patients.

### 4.2. Multiple Degrees-of-Freedom Gait Rehabilitation Motion Control Verification of Scissor Mechanism with Lower-Limb Exoskeleton Robot

**Figure 14:**
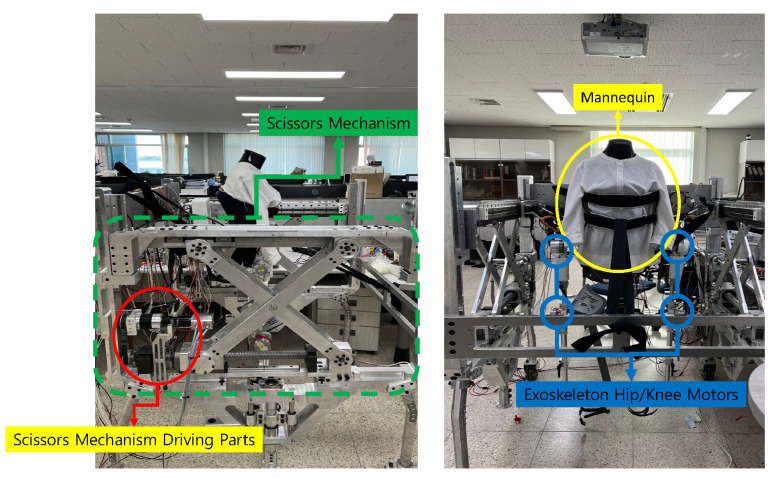
Experiment setup. The left figure shows the side of the pelvic support robot. The green dotted line indicates all the scissor mechanism parts. The red circle indicates the scissor mechanism driving parts. This motor facilitates the pelvic obliquity motion. The right figure shows the front side of pelvic support robot with the exoskeleton robot. We use the mannequin as a human dummy (indicated by the yellow circle). The four blue circles indicate the exoskeleton robot motors. These motors facilitate the hip/knee flexion and extension motion.

Experiments were conducted to verify that the scissor mechanism and the exoskeleton-integration system functioned to provide the desired motion. Because the scissor mechanism facilitates pelvic obliquity motion and the exoskeleton assists the hip/knee flexion/extension motion, each robot was made to replicate a human walking motion to verify this. The main controller of the system comprised an NI-Sbrio 9627 model. The scissor mechanism motors were of the MAXON EC 45 BLDC 250-W model, and the motors of the exoskeleton used the Kollmorgen RBE-01510B and RBE-01212C models, respectively. The ELMO WHI 10/100 model was used as a motor driver for controlling each motor. The robot used can communicate at 1 kHz. The HEDL 9140 ENCODER was used to measure the motion of the robot, and the resolution of the encoder was 500 counts per turn. The proportional integral derivative controller of the motor tuned the parameters by using the auto-tuning function of the ELMO motor controller. As shown in Figure 14, the experiment was conducted by using a mannequin with an exoskeleton robot.

**Figure 15:**
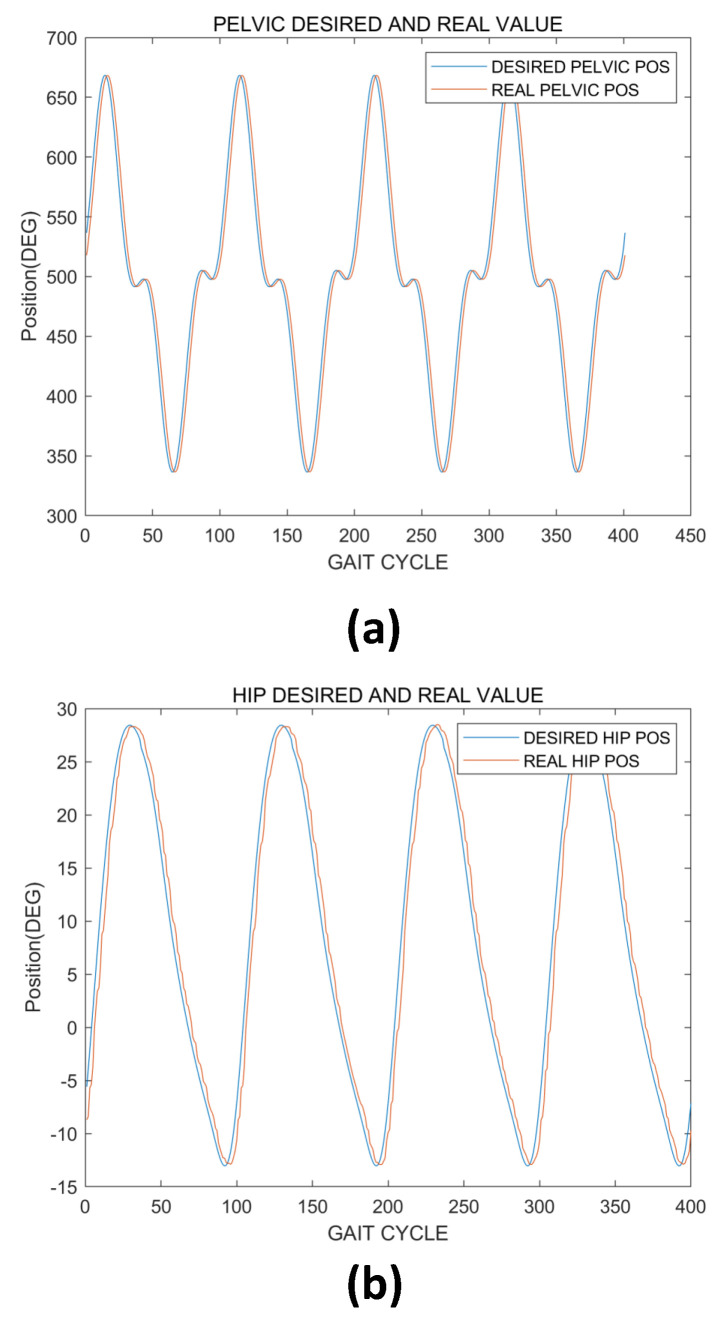

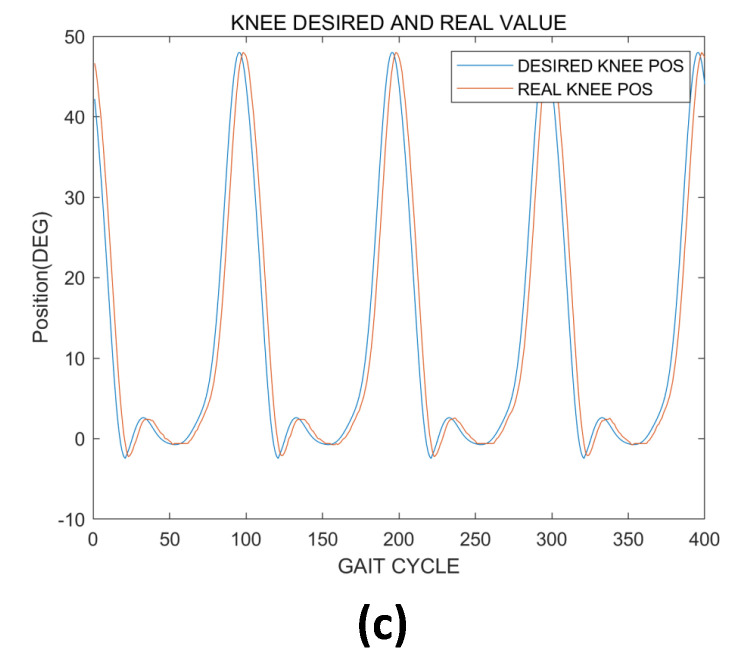
Experiment results. (**a**) Desired pelvic obliquity motion motor position command (indicated by blue line) and real value (indicated by red line). (**b**) Desired hip flexion/extension motion position command (indicated by blue line) and real value (indicated by red line). (**c**) Desired knee flexion/extension motion position command (indicated by blue line) and real value (indicated by red line). These three graphs show that the system can follow the human gait motion with little delay.

The experimental results are as follows. Figure 15 presents the results of the desired motion and the resulting real value of the robot’s movement when the scissor mechanism and exoskeleton robot are given motion commands, respectively. The results showed that the scissor mechanism’s motor for facilitating pelvic motion and the exoskeleton’s motor that produces hip/knee motion in the sagittal plane follow the desired motion well (Hip/Knee Reference human motion data resource from Hanyang University HARCO LAB). The results showed that the pelvic support mobile-type robot could, on paper, generate harmonious motion with the exoskeleton and be used for gait rehabilitation in patients.

## 5. Conclusions and Future Work

In this study, we developed a pelvic support robot by using a scissor mechanism. By using the scissor mechanism on both sides of the patient, pelvic obliquity motion was realized in order to develop a system that can support the rehabilitation plane and not only in the sagittal plane, as in the existing walking rehabilitation exoskeleton robot. In this study, we evaluated the H/W validation with CAE analysis, and a test was conducted by integrating the exoskeleton systems of previous research with a pelvic support robot. CAE analysis showed that the system can support BWS force and pelvic assist force under 100-kg patients by the scissor mechanism. The motion of the system was tested by using human gait motion, as shown in the results in Figure 15. The system can generate the harmonious gait motion by integrating the pelvic support and exoskeleton robots. Therefore, during walking training, pelvic motion was possible, and accurate gait-rehabilitation training motion can be achieved by using our system.

We finished our system evalulation without using a human dummy. In future work, the human experiment will be performed to investigate the effectiveness of gait rehabilitation on the proposed system through a comparison of the pelvic motion between healthy subjects and stroke patients.

## Figures and Tables

**Table 1:** The simulation parameters for calculating force $F_{1}$.

Parameters	$\theta_{s}$	m	g
Value	$10^{\circ }∼90^{\circ }$	100 kg	9.8 m/s${}^{2}$

## Data Availability

Hip/Knee Reference human motion data resource from Hanyang University HARCO LAB (https://hyharco.github.io/team/).
